# Assessing Disease Severity in Common Variable Immunodeficiency Disorders (CVID) and CVID-Like Disorders

**DOI:** 10.3389/fimmu.2018.02130

**Published:** 2018-09-28

**Authors:** Rohan Ameratunga

**Affiliations:** ^1^Department of Virology and Immunology, Auckland City Hospital, Auckland, New Zealand; ^2^Department of Clinical Immunology, Auckland City Hospital, Auckland, New Zealand

**Keywords:** CVID, LOCID, CVID-like, CDSS, epistasis, digenic, PID

## Introduction

Common Variable Immunodeficiency Disorders (CVID) are the most frequent symptomatic primary immune deficiency (PID) in adults. Current estimates suggest a prevalence of ~1:25,000, although a recent study has suggested an even greater frequency than previous estimates (1). The majority of CVID patients suffer recurrent infections because of late onset antibody failure (LOAF) leading to immune system failure (ISF). Current criteria do not allow CVID to be diagnosed before 4 years although some patients have symptoms dating back to infancy. Most patients experience recurrent or severe bacterial infections and less commonly autoimmunity as a result of CVID. Some patients present with a sarcoidosis-like disorder or enteritis (2, 3). A proportion of patients with CVID have a prominent T cell defect leading to severe viral or opportunistic infections. These patients have been deemed to have late onset combined immunodeficiency (LOCID) (4). LOCID is currently separated from CVID although I have argued LOCID should be included as a subset of CVID (5).

In spite of major progress in the last decade, the genetic basis of CVID is unknown in most patients. A causative mutation has been identified in up to 30% (6). If a causative defect is identified, these patients are removed from the umbrella diagnosis of CVID and are reclassified as having a CVID-like disorder caused by a specific mutation. To fulfill a diagnosis, all current CVID criteria require exclusion of other immunodeficiencies including *NFKB1, NFKB1, CTLA4* etc. It is however likely earlier series of patients with CVID included many with CVID-like disorders, whose mutations were undiscovered.

**Figure 1:**
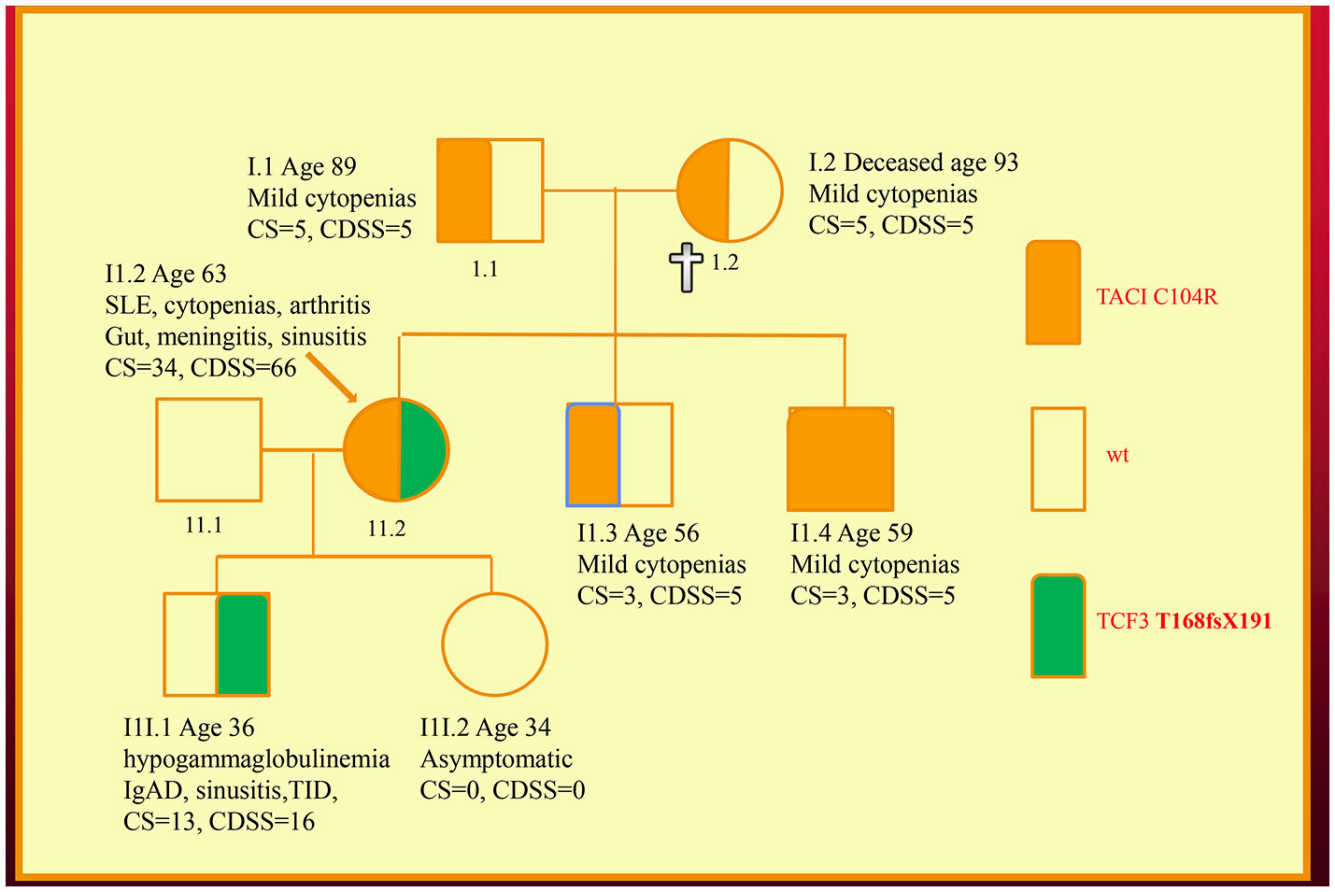
Family with a digenic CVID-like disorder caused by epistatic interactions of *TNFRSF13B*/TACI and *TCF3* genes. The proband (arrow) suffers from both a severe immunodeficiency as well as SLE. Other members are as described in our previous publications including a mild symptomatic brother (II.3) with severe hypogammaglobulinemia caused by homozygous C104R mutations of the *TNFRSF13B*/TACI gene. CDSS, CVID disease severity score; CS, clinical score. The CS was suggested as means of determining eligibility for SCIG/IVIG but we have used it as a surrogate marker of disease severity (5, 7, 11).

We have recently discovered new genetic defects in two NZ families with CVID-like disorders. In the first family, we have confirmed the existence of quantitative epistasis in humans (7). Epistasis is the non-linear, synergistic interaction of two or more genetic loci either leading to a much more severe disorder or a novel phenotype (8, 9). The existence of epistasis was first predicted by William Bateson in 1909 but has remained highly controversial because of the lack of well characterized examples in humans (10). In this family, the synergistic interaction of *TNFRSF13B*/TACI and *TCF3* mutations resulted in a severe immunodeficiency and systemic lupus erythematosus (SLE) in the proband (Figure 1). Other members of the family who have various permutations of the two mutated genes had a milder phenotype, which was reflected in their *in vitro* B cell differentiation and antibody production studies (7).

**Figure 2:**
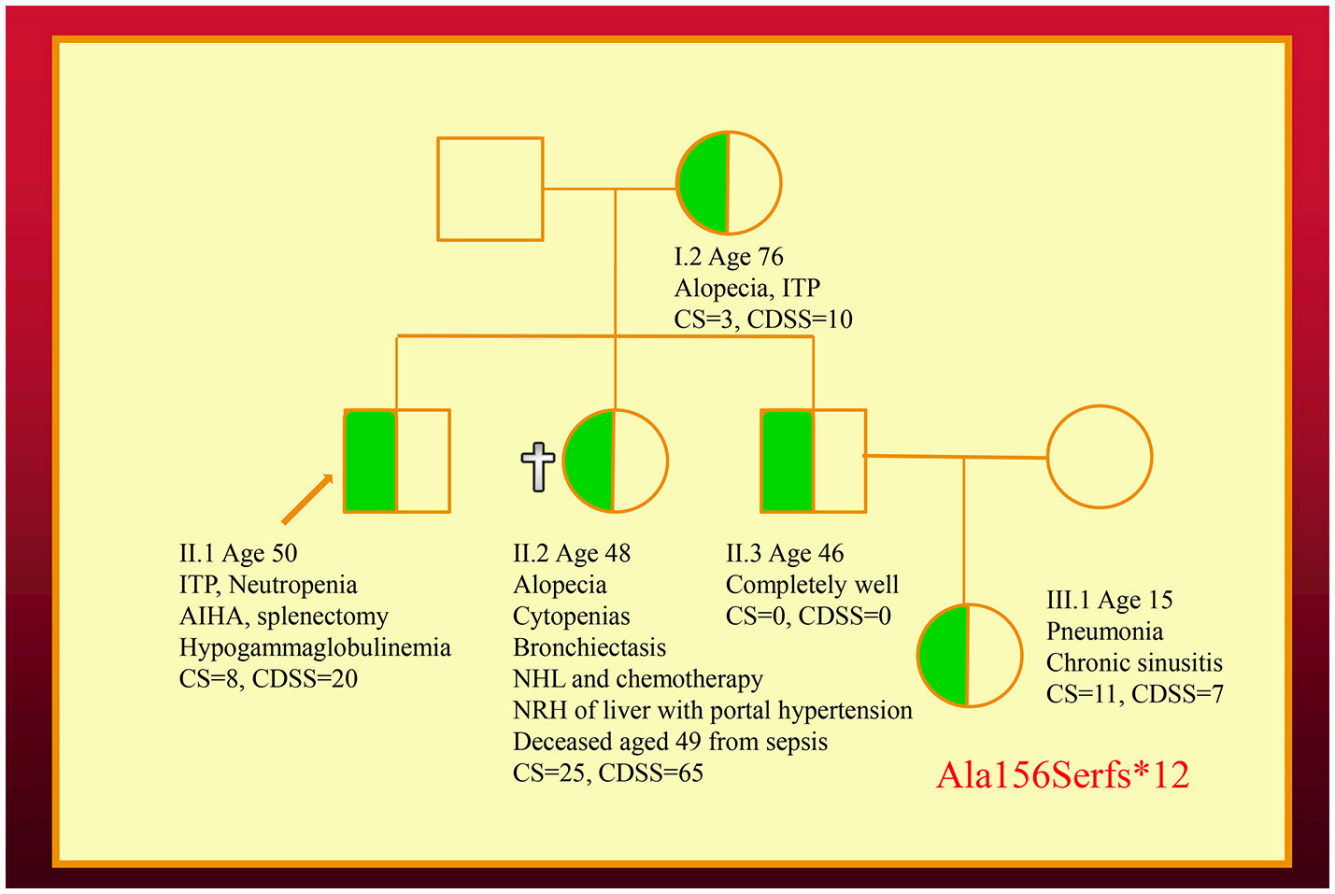
Family with an *NFKB1* mutation. The proband (II.1) is shown with an arrow. Other family members are described in the text. Note that siblings of III.1 are not shown. CDSS, CVID disease severity score; CS, clinical score.

We have also co-discovered *NFKB1* mutations as a cause of a novel CVID-like disorder in the second family (Figure 2) (12). It was striking there was a very broad spectrum of phenotypes in this NZ family in spite of carrying the identical mutation. The recently deceased sister had a severe LOCID phenotype, while a 46-year-old asymptomatic brother carries the identical mutation (12). Other members of the family have widely varying phenotypes including recurrent infections or autoimmunity (5).

Here I present a disease severity score for CVID and CVID-like disorders (Table 1). There are potentially many clinical and theoretical advantages in developing a disease severity score in CVID and CVID-like disorders (Table 2). A very high score might suggest the presence of epistasis or LOCID. A diagnosis of LOCID will require confirmatory laboratory tests. A disease severity score may have clinical utility in allowing closer follow-up of severely affected patients in clinics and offering prognostic information. These potential advantages are discussed in more detail below.

**Table 1:** An instrument for assessing CVID disease severity.

**Parameter**	**Mild = 1**	**Moderate = 5**	**Severe = 10**	**Score/date**
CNS		Asymptomatic MRI changes, viral meningitis with no sequelae	Meningitis, CNS granulomatous or lymphocytic vasculitis, Cauda equina syndrome, Other CNS autoimmune disorders incl MS, Peripheral neuropathy including CIDP, Echovirus encephalitis, ^*^Cryptococcal meningitis, etc.	
Ocular		Uveitis responding to treatment	Sight threatening disease, e.g., keratitis, retinopathy, or retinal vasculitis	
ENT/ORL	Otitis media, acute sinusitis, otitis externa	Chronic rhinosinusitis	Complicated mastoiditis (e.g., hearing loss, intracerebral sepsis) Autoimmune hearing loss	
Pulmonary	Mild asthma, uncomplicated pneumonia	Mild GLILD, mild bronchiectasis, moderate-severe asthma, complicated pneumonia	Severe pulmonary dysfunction based on lung function tests, Extensive bronchiectasis, Severe GLILD, lung surgery (not biopsy). Pulmonary hypertension, Lung transplantation, Chest infections due to Pseudomonas^*^PJP,	
Cardiac		Pericarditis	Coronary vasculitis, myocarditis, cardiac transplantation, endocarditis,	
Gut/nutrition	Oral ulceration or glossitis responding to treatment, oral candidiasis, Giardia, or Helicobacter pylori responding to treatment, Uncomplicated Vitamin or mineral deficiency	Mild IBD responding to budesonide, cholecystitis, celiac disease, AI gastritis, severe infectious enteritis, complicated vitamin or mineral deficiency.	Severe IBD requiring immunosuppression, severe enteritis, peritonitis, severe malabsorption incl protein-losing enteropathy, unresponsive norovirus infection, Severe malnutrition e.g., BMI < 18, or failure to thrive (children)	
Liver	Asymptomatic increase in liver enzymes.	Mild NRH, autoimmune hepatitis/granulomatous or viral hepatitis responding to treatment. Portal hypertension on imaging.	NRH with cirrhosis and/or symptomatic portal hypertension, Complicated/ unresponsive viral hepatitis. Liver transplantation. Severe AI hepatitis. Primary biliary cirrhosis,	
Spleen	Asymptomatic splenomegaly	Splenectomy-(risk of sepsis) Symptomatic splenomegaly		
Renal	Uncomplicated UTI's	Granulomatous involvement of urinary tract on imaging	Chronic renal failure from e.g., renal vasculitis or granulomatous disease. Renal transplantation.	
Hematological	Mild asymptomatic cytopenias,	Requiring treatment	Life threatening/Poorly responsive cytopenias e.g., requiring splenectomy or rituximab, HSCT	
Lymph nodes Non-malignant	Mild lymphadenopathy	Extensive incl sarcoid-like granulomatous disorder		
Musculoskeletal	Arthralgia, myalgias, mild osteopenia Mycoplasma/ ureaplasma arthritis responding to treatment	Arthritis, other treatment responsive CTDs, myositis, severe osteoporosis,	Osteomyelitis, Severe CTDs e.g., requiring biologicals,	
Vasculitis		Cutaneous	Systemic	
Endocrine	Autoimmune thyroiditis	Addison's disease, ACTH deficiency, diabetes insipidus	Hypophysitis, T1D	
Cutaneous	HSV1 cold sores, mild cellulitis, chronic urticaria,	Extensive VVC, uncomplicated shingles, Psoriasis, lichen planus, ^**^Alopecia, Vitiligo	Pyoderma gangrenosum	
Malignancy			Present (CVID associated)	
Other infections	^*^Uncomplicated EBV or CMV viremia	Non-life threatening abscesses,	Sepsis, life-threatening abscesses. ^*^CNS EBV/CMV lymphoproliferative disease, ^*^disseminated fungal infection. ^*^Disseminated adenovirus infection	
Other autoimmunity	Uncomplicated pernicious anemia,	Sjogren's syndrome, anti-IgA antibodies. Cutaneous lupus	Severe SLE, APLS,	
“Allergies” (including non-allergic conditions)	Rhinitis, mild eczema	Severe eczema, food allergies, Multiple antibiotic allergies Reactions to SCIG/IVIG		
Iatrogenic complications		Complications from long term steroids	Life-threatening complications e.g., CSF leak following sinus surgery. Hepatitis C from IVIG, complications from organ transplantation and HSCT, severe complications from immunosuppression	
Misc and rare			Amyloidosis, HLH	
Sundry				

*CDSS, Cephalo-caudal and based on organ system affected. Individual patient scores can be entered on the right hand columns of the Table. The total score can be computed from each organ system. The right hand column allows chronological documentation of cumulative disease burden. AI, autoimmune; APLS, antiphospholipid syndrome; BMI, body mass index; CIDP, chronic inflammatory demyelinating polyneuropathy; CTD, connective tissue disorder; GLILD, granulomatous lymphocytic interstitial lung disease; HLH, Hemophagocytic lymphohistiocytosis; HSCT, hematopoietic stem cell transplantation; HSV, Herpes Simplex Virus; IBD, inflammatory bowel disease; MS, multiple sclerosis; NRH, nodular regenerative hyperplasia of the liver; PJP, Pneumocystis jirovecii pneumonitis; SLE, systemic lupus erythematosus; TID, type 1 diabetes; UTIs, urinary tract infections; VVC, vulovaginal condylomatosis*.

* *I acknowledge some experts would not include patients with severe fungal, viral and opportunistic infections in the broad spectrum of CVID*.

** *Although not life-threatening, alopecia, and vitiligo, particularly of the face have the potential to cause severe psychological damage from body image distortion*.

**Table 2:** Potential advantages of a disease severity score for CVID.

**Potential advantages of a disease severity score for CVID**
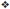 Potentially identifying patients with LOCID
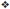 Identifying possible instances of epistasis
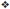 Prognosis including mortality risk
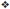 Assessing disease severity in individual patients
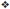 Assisting with disability claims
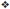 When to commence SCIG/IVIG in CVID-like disorders
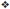 Case mix for different clinicians
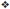 Comparing CVID cohorts
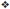 Comparing CVID groups in randomized trials e.g., SCIG/IVIG

*Future studies will indicate if these advantages are validated*.

There has been a previous attempt at generating a disease severity score seven years ago (13). Here I develop these ideas further by focusing on target organ damage, which has been shown to determine prognosis. Because there is disagreement about the precise laboratory values which define CVID, (14) these have not been included in the CVID disease severity score (CDSS) in Table 1. Qualifying laboratory values or genetic mutations could be added if this disease severity instrument is to be used in a patient registry. To illustrate its utility, the CVID disease severity scores of the two families are presented here and compared with the clinical score.

## The CVID disease severity score (Table 1)

The CDSS focuses primarily on cumulative organ damage as a result of infections, autoimmunity or inflammation. All of these are examples of ISF, as they indicate the presence of immunodeficiency or dysregulation of the immune system. Most of the sequelae (Table 1) quantified in the CDSS have been described in large cohorts of CVID patients, which as noted above, likely also included CVID-like patients (3, 15–19). It thus seems reasonable to apply this instrument to patients with CVID-like disorders also. To simplify the instrument, the severity of complications have been arbitrarily divided into three categories, mild, moderate, and severe. In general, mild manifestations can be easily treated and do not cause long-term morbidity. Moderate category conditions do cause short and long-term morbidity and may not be reversible. Conditions falling into the severe category are either life threatening or have the potential to cause severe disability such as visual loss or severe pulmonary dysfunction.

There is probably general agreement amongst experienced colleagues that some complications of CVID and CVID-like disorders are more severe than others (20). There is also support from previous studies indicating complications such as enteritis, malnutrition, cytopenias, and malignancy have a worse prognosis in patients with CVID (3, 18). Mortality is also increased in patients with functional or structural lung disease or hepatitis (16, 21). These have been placed in the severe column. In contrast, the worse prognosis with delayed diagnosis and early onset disease is likely to be reflected in higher organ damage scores in the CDSS. Early onset of disease may indicate a more aggressive disease trajectory while delay in diagnosis is likely to result in more severe organ damage from unmitigated infection and inflammation (3, 16).

As noted above, it is possible for patients to change categories if they deteriorate. A patient with mild bronchiectasis can progress to the severe category if the bronchiectasis becomes more extensive. Mild bronchiectasis is defined as causing few or no symptoms but is demonstrated on CT scans. I have not attempted to define the precise severity of bronchiectasis based on the number of lobes affected or extent of damage. If a patient has more than one complication in an organ system, each complication will receive a separate score e.g., a patient who has both severe bronchiectasis and severe interstitial lung disease will have a score of 20 (10+10). Similarly, if a patient suffers peritonitis, it is recorded only once in the GI complications list but not as an additional severe infection.

Recurrent uncomplicated pneumonias are not scored, as these are likely to be a reflection of underlying bronchiectasis, under treatment with SCIG/IVIG, chronic upper respiratory tract disease (22) or due another condition such as undiagnosed gastroesophageal reflux (20). In the absence of these predisposing factors, future studies will indicate if this group should be included in the moderate category. Similarly, recurrent acute otitis media and recurrent acute sinusitis are likely to reflect chronic rhinosinusitis.

Malignancy related to CVID was excluded from our diagnostic criteria for CVID, as it can be difficult to determine if malignancy is the cause or the result of CVID (23, 24). It has been included in this disease severity score, as its presence will clearly influence prognosis (25). Similarly, damage to some organ systems such as the CNS are unlikely to result in mild complications, most are likely to be life-threatening or have the potential to cause severe disability.

I have not included numbers of antibiotic courses, as this may vary with local practice. Some centers administer prophylactic antibiotics routinely. I have included Pseudomonas lung infections as this is often seen late in the course of CVID lung disease and is very difficult to treat. It is likely to be an important prognostic marker (22).

I have also included multiple antibiotic allergies as a moderate complication, as this will limit treatment options and therefore adversely affect prognosis (26). Patients are also at increased risk of morbidity if they have to be desensitized acutely to antibiotics. Total IgE is decreased in the majority of CVID patients (27). Asthma and rhinitis in the absence of bronchiectasis and chronic sinus disease however, appear to feature prominently in some series of CVID patients and may be an intrinsic part of the immune dysregulation of CVID.(28, 29) Most, (but not all) have negative skin tests or specific IgE to common aeroallergen (26).

Splenectomy appears to be surprisingly well tolerated in CVID patients and has been placed in the moderate category (30). The main prognostic issue is the risk of sepsis. Note that precise values for the sizes of an enlarged spleen and lymph nodes were suggested in a previous scoring system. I have avoided this, as current prognostic data does not appear to support such precise cut-offs at this time (13). Perhaps future cohorts may validate such precise approaches and the scoring system could be changed.

Treatment of CVID can lead to complications and this has been included in the disease severity score. In some cases treatment can lead to life-threatening sequelae such as CSF leaks following endoscopic sinus surgery or Hepatitis C from IVIG preparations. Iatrogenic complications have been kept separate, as it allows a clinician to discuss future therapy in the context of problems from previous treatment. Complications from solid organ transplantation and hematopoietic stem cell transplantation (HSCT) have been placed in this category. The CDSS may be useful in determining if there has been a decrease in disease severity following HSCT for CVID. Some complications may resolve while others may emerge after HSCT.

The CDSS has not included unrelated co-morbid conditions such as severe (atherosclerotic) coronary artery disease, which will clearly affect an individual's prognosis.

The recognized phenotypic spectrum of CVID (based on diagnostic criteria) and CVID-like disorders (based on the mutation) are expanding (8). The last row has been intentionally left blank for rare or new manifestations of CVID/CVID-like disorders. This will give some flexibility so new manifestations can be included in the future. It will also mean the maximum score for the CDSS may change over time. Any future studies using this instrument should define new criteria placed in the last row.

## Discussion

Disease severity instruments vary in their utility. Some instruments such as injury severity scores accurately predict mortality following trauma (31). These instruments are however less able to predict the severity of long-term disability in survivors of major trauma. Often, disease severity instruments evolve over time when new information becomes available from prospective studies. This is seen in SLE, where there have been multiple iterations of the original SLEDAI (SLE Disease Activity Index) and BILAG (British Isles Lupus Assessment Group) scoring systems (32).

The CDSS can be used either as an index of disease burden or an index of disease activity in CVID/ CVID-like disorders, where scores improve when complications such as ITP or AIHA are treated and are no longer active. I suggest however that the scores are added as an index of cumulative disease burden. This is more likely to reflect the prognostic trajectory of a patient with CVID. In rare CVID/ CVID-like patients with predominant autoimmune disease, it may be useful as a disease activity score, analogous to the SLEDAI.

There are thus competing goals when formulating disease severity scores. The table I have presented can be used clinically and can be updated on a regular basis, particularly if a patient's disorder is in evolution. Each complication can be dated in the right hand column to provide a comprehensive overview of an individual patient's progress over time.

The CDSS will be useful in busy clinics where the same patient is reviewed by different clinicians on separate visits. It may alert a new clinician to the likely severity of the CVID/ CVID-like disorder in an individual patient. It may help achieve consistency in how often patients are followed up in clinic. In general, patients with complex disorders or those rapidly deteriorating require close clinical supervision.

The CDSS will assist with determining the case mix of a clinician in a Clinical Immunology department. This will help with job sizing, as it will offer an objective assessment of the complexity of each clinician's workload. It will also serve as a check-list for junior colleagues in training. When a patient with CVID is seen for the first time, damage to each target organ should be carefully assessed. It may also be useful comparing disease burden in control vs. treatment arms in future randomized trials of new SCIG/IVIG products. It may help identify allocation bias when assessing outcomes of different treatments.

As I have shown here, a disease severity score is very useful in family studies. The CDSS correlates with the clinical phenotypes in both kindreds (Figures 1, 2). As can be seen in the first family, the digenic proband has the highest CDSS, consistent with the epistatic interactions of the *TNFRSF13B*/TACI and *TCF3* mutations (7). Her son carrying only the *TCF3* mutation has a higher score than other family members bearing either homozygous or heterozygous mutations of the *TNFRSF13B*/TACI gene. This indicates the *TCF3* mutation has a much greater impact on disease severity compared to mutations of *TNFRSF13B*/TACI (9).

In this digenic family, the CDSS supports the separation of genes predisposing to CVID vs. those causing CVID-like disorders. In our diagnostic criteria, we have separated genes which cause CVID-like disorders (*CTLA4, LRBA, NFKB1* etc.) from those which predispose to CVID (*TNFRSF13B*/TACI, *BAFFR, TWEAK, MSH5*) (23). As seen in the first family, there are substantial differences in the disease severity scores of family members bearing *TNFRSF13B*/TACI vs. *TCF3* mutations, showing effective genotype-phenotype separation (9). If patients carrying genes predisposing to CVID have a high CDSS, this should prompt a search for a second causative mutation as seen in the first family.

In the second kindred, the proband's sister (II.2) with the LOCID sub-phenotype had the highest score compared with other family members (5). This reflects the severe damage to multiple organ systems caused by the T cell defect. A high CDSS should alert a clinician to the possibility of the LOCID sub-phenotype of CVID. A disease severity instrument can thus be useful in providing prognostic information (Table 2). Patients with multiple category 3 complications have a more severe disease burden and are likely to have an increased risk of mortality. Patients rapidly developing multiple complications may be candidates for alternative forms of treatment including HSCT (33, 34).

This instrument can thus be applied to patients with CVID-like disorders also, where a causative mutation is identified (8). Both CVID and CVID-like disorders appear to share analogous subphenotypes, such as the autoimmune variant or the infections only phenotype (5). In CVID-like disorders, the CDSS can be used to monitor asymptomatic family members carrying the same mutation. If they start developing symptoms, they may be candidates to begin SCIG/IVIG.

In the future, the CDSS could also be used to compare the severity of different mutations causing CVID-like disorders. Because of the effects of variable penetrance and expressivity, large cohorts of CVID-like patients will be needed to compare the CDSS of different mutations. Furthermore, age of onset and disease duration will need to be factored when comparing CDSS scores for different mutations. The precise location of the mutation might influence the phenotype. As seen in the brother (II.3) in the second family, it is becoming apparent that a proportion of patients carrying mutations of CVID-like disorders are asymptomatic (8). In contrast to patients bearing mutations of genes predisposing to CVID (*TNFRSF13B*/TACI, *BAFFR, TWEAK, MSH5*), the CDSS is likely to vary widely in patients bearing mutations of CVID-like genes.

Such a disease severity instrument can also be useful in supporting insurance and disability claims and also show the need for ongoing social support of these patients. Having a disease severity score may assist funders of SCIG/IVIG, as was originally proposed for the clinical score (11). Given the difficulties reliably assessing vaccine responses in CVID patients, (35, 36) clinical symptoms may be a more reliable marker of LOAF/ISF, as it reflects ISF (14).

This instrument may also be useful in dealing with patients with primary hypogammaglobulinemia who have not met indications for SCIG/IVIG replacement in a particular clinic. We have divided these patients into symptomatic (sHGUS) vs. asymptomatic (aHGUS) hypogammaglobulinemia of uncertain significance (23). In our practice symptoms are a major indication for SCIG/IVIG treatment, providing the patient also meets laboratory criteria for CVID (35).

For comparison, both the CDSS and the CS are shown in the family pedigrees (Figures 1, 2). In both of the families there appears to be close correlation between the CDSS and CS. The CDSS however covers many more affected organ systems than the CS, which is similar to a previously described list of 15 “unlucky complications” of CVID (13, 37). The CDSS may therefore be more sensitive than the CS or the list of 15 “unlucky complications.”

There are important caveats to any disease severity instrument (Table 3). There may be some intra-observer variability for example when scoring the severity of bronchiectasis. It is more likely there will inter-observer variability. Some clinicians may judge a complication as moderate, while others may assess it as severe. This may be less of an issue for an individual patient than when comparing patients. I also acknowledge there is heterogeneity within these complications: some patients with GLILD may respond to IVIG alone, while others may require more intense therapy. With better methods to quantify organ damage and response to treatment, a finer scale may provide reproducible data in the future.

**Table 3:** Disadvantages of a disease severity score for CVID.

**Limitations of a disease severity score for CVID**
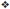 Disease burden vs. disease activity
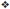 Absolute score may not reflect severity of condition
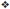 Is not a diagnostic tool for CVID
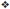 Does not address Quality Of Life e.g., fatigue
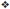 Intraobserver variability i.e., consistency
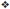 Interobserver variability e.g., determining severity of bronchiectasis
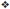 Does not address heterogeneity of severity within complications
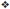 Does not reflect response to treatment in each complication
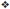 Score may not identify different patterns of organ systems damage when comparing international CVID cohorts

*See text for full discussion*.

The absolute score may not necessarily reflect the severity of the condition in any given patient. Patients with chronic rhinosinusitis and treated ITP for example will have the identical score as a patient with end stage lung disease from bronchiectasis. Similarly, in the absence of sequelae, patients with otitis externa receive the same score as a patient with uncomplicated pneumonia. As noted above, patients with multiple severe complications (column 3) are however likely to have a higher disease burden with increased morbidity and mortality risk. This is seen in both the families presented here.

When comparing international cohorts, it may be more informative to compare scores for each complication. The absolute score may mask important differences including rates of bronchiectasis vs. autoimmunity etc. Such differences have been demonstrated in different CVID cohorts across Europe (3). It is important to note the CDSS cannot be used as diagnostic criteria for CVID. It is a disease severity score for patients with hypogammaglobulinemia, CVID and CVID-like disorders. Diagnostic criteria for CVID have been previously discussed and the reader is referred to these publications (14, 23, 38, 39).

This instrument does not address Quality of Life (QOL) in CVID. Fatigue is an important symptom affecting CVID patients but is not included in this instrument (40). Several studies have used established QOL surveys or have developed new instruments for patients with CVID and antibody deficiency (41–43). The disease severity score presented here could be complemented by the SF36 questionnaire or similar QOL instruments.

The strengths and weaknesses of this instrument will become apparent over time. It will be important to validate the CDSS with long-term prospective cohorts from around the globe. This will be helpful in assessing various aspects of validity and reliability of this instrument (32). I would be very pleased to receive feedback and suggestions for improving future iterations of this CVID disease severity instrument.

## Ethics statement

This manuscript complies with ethics standards. All studies have been undertaken with the consent of both families. These studies are approved by the NZ Ministry of Health Ethics committee and the ADHB ethics committee. There are no ethical impediments to publish this work.

## Author contributions

The author confirms being the sole contributor of this work and has approved it for publication.

### Conflict of interest statement

The author declares that the research was conducted in the absence of any commercial or financial relationships that could be construed as a potential conflict of interest.
